# Gastrointestinal histological injury in pigs subjected to triple stent interposition in the thoracoabdominal aorta

**DOI:** 10.1590/acb402425

**Published:** 2025-03-10

**Authors:** Angela Claudia Paixão Soares de Magalhães, Gutenberg do Amaral Gurgel, Svetlana Maria Wanderley de Barros, Miguel Lucas Silva Valente, Maurício de Amorim Aquino, Sthefanie da Silva Bessa, Rogério Ferraz Baquette, Aldemar Araújo Castro, Guilherme Benjamim Brandão Pitta

**Affiliations:** 1 Universidade Federal do Acre – Faculdade de Medicina – Departamento de Cirurgia – Rio Branco (AC) – Brazil.; 2 Universidade Federal do Rio Grande do Sul – Faculdade de Medicina – Departamento de Cirurgia – Porto Alegre (RS) – Brazil.; 3 Centro Universitário São Lucas – Faculdade de Medicina – Departamento de Cirurgia – Porto Velho (RO) – Brazil.; 4 Fundação Hospital Estadual do Acre – Rio Branco (AC) – Brazil.; 5 Universidade Estadual de Ciências da Saúde de Alagoas – Faculdade de Medicina – Departamento de Cirurgia – Maceió (AL) – Brazil.; 6 Universidade Estadual de Ciências da Saúde de Alagoas – Faculdade de Medicina – Departamento de Cirurgia – Maceió (AL) – Brazil.

**Keywords:** Aorta, Stents, Wounds and Injuries, Endovascular Procedures, Models, Animal

## Abstract

**Purpose:**

To evaluate gastrointestinal histological injury in pigs subjected to triple stent interposition *versus* a control group, hypothesizing no significant injury increase with triple stents.

**Methods:**

A prospective study with 15 pigs divided into a control group (G0, n = 5) undergoing arteriography only, and a triple stent group (G3, n = 10) undergoing arteriography and three stent implantations in the thoracoabdominal aorta. After an eight-day observation, arteriography, euthanasia, and en bloc gastrointestinal harvesting were performed. Lesions were graded using the Park/Chiu classification, and serum markers were analyzed pre- and post-procedure.

**Results:**

Arteriography confirmed mesenteric artery patency in all animals. Histological analysis showed ischemic lesions in 88.9% of G3, mainly in the colon (89%), compared to 60% in G0, primarily in the colon (60%) and stomach (40%). Most G3 lesions were grade 1, while G0 had higher-grade lesions. Serum markers showed no significant intergroup differences.

**Conclusion:**

Triple stent interposition did not significantly increase gastrointestinal injury, indicating its safety for maintaining gastrointestinal perfusion in this model.

## Introduction

Intestinal ischemia is a significant complication in thoracoabdominal aorta surgeries, with an estimated incidence of 2.5% and a 62% mortality rate^1^. Even with advanced techniques such as reimplantation of the superior mesenteric artery and maintenance of the patency of the internal iliac arteries, gastrointestinal hypoperfusion can occur, contributing to considerable postoperative morbidity and mortality^2^.

Endovascular treatment has emerged as a less invasive alternative, associated with reduced morbidity and mortality compared to open surgery^3^. The interposition of multiple stents, such as the triple stent, aims to maintain blood flow to the visceral branches while treating complex aortic lesions^4^^–^ 6. Studies involving polymer-coated and drug-eluting stents have shown complete endothelial coverage and reduced intimal hyperplasia, which are essential for maintaining perfusion and preventing ischemia^7^.

However, the safety of this technique regarding the integrity of intestinal tissues still requires evaluation. The possibility of mesenteric hypoperfusion resulting from the implantation of multiple stents in the thoracoabdominal aorta raises concerns about the risk of intestinal ischemia. Therefore, this study sought to determine whether gastrointestinal histological injury in pigs subjected to triple stent interposition differs significantly from those in a control group (arteriography only), thus contributing to the understanding of the safety and efficacy of this therapeutic approach.

## Methods

This study was a prospective, randomized experimental trial conducted at the Experimental Surgery Center of Braile Biomédica, São José do Rio Preto, SP, Brazil. The study protocol was approved by the Animal Use Ethics Committees of the Universidade Estadual de Ciências da Saúde de Alagoas, under protocol 113A, and Braile Biomédica under protocol 02/2015, in accordance with the guidelines outlined by the Brazilian College of Animal Experimentation, Federal Law no. 11.794/2008, and Resolution no. 714/2002.

A total of 15 hybrid pigs (Landrace × Large White), aged between 8 and 10 weeks old and weighing 15–20 kg, were used. The inclusion criteria required animals free of diseases and any morphological alterations of the aorta and its branches. The pigs were randomly assigned to two groups:

- Control group (G0, n = 5): underwent only arteriography (no stent placement);
- Triple stent group (G3, n=10): underwent arteriography and triple stent implantation in the thoracoabdominal aorta.

**Figure 1:**
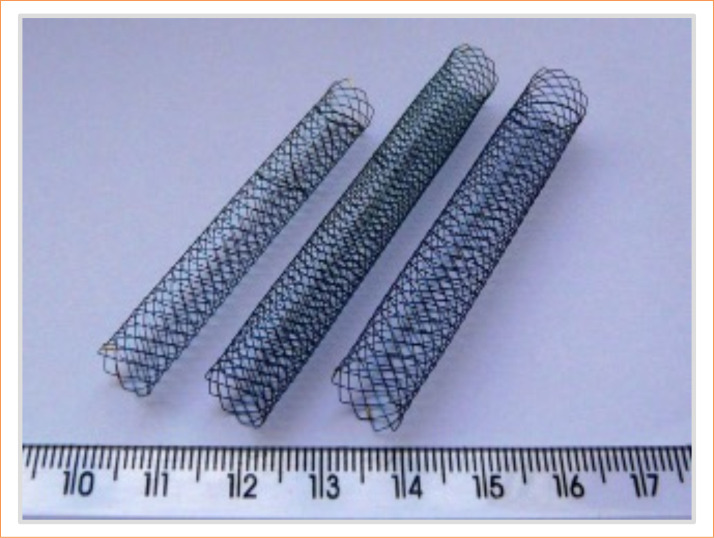
Design of stents.

Source: Adapted from Barros et al.^8^.

Randomization was carried out using block permutation, ensuring equal allocation between groups. Figure 1 presents the design of the stents used.

### Anesthesia and surgical procedures

Prior to surgery, animals underwent a 12-hour fasting period for solids and a 3-hour fasting period for water. Premedication consisted of atropine (0.02 mg/kg, SC) to reduce secretions, followed by sedation with ketamine (10 mg/kg, IM) and midazolam (0.2 mg/kg, IM)^9^. After sedation, intravenous access was obtained via the marginal ear vein, and general anesthesia was maintained with halothane (1–2%) in 100% oxygen using a face mask.

Under aseptic conditions, the right carotid artery was surgically exposed, and arterial access was achieved with a 16G Jelco needle under direct visualization. A 0.035-inch hydrophilic guidewire was introduced under fluoroscopic guidance, followed by insertion of a 7F/11-cm vascular sheath. An initial arteriography was performed using a 5F/100-cm multipurpose catheter to assess the aortic anatomy and branches.

### Stent implantation

In the G3, three self-expanding nitinol stents (Braile Biomédica), featuring a closed-cell design and gold radiopaque markers, were deployed sequentially in the thoracoabdominal aorta, covering from just distal to the left subclavian artery down to the iliac bifurcation. Overlapping of approximately 10 mm was ensured between stents to maintain continuous coverage.

In the G0, no stent was implanted; only the initial arteriography was performed. The average procedural time for G3 was 49 minutes (range: 25–90 minutes), and for G0, 39 minutes (range: 30–55 minutes).

### Postoperative care and euthanasia

After the procedure, all animals were kept in individual pens with free access to water and food for eight days. During this observation period, they were monitored daily for any complications, such as discomfort, infection, or signs of ischemia.

On the eighth day, under anesthesia induced as described previously, a final arteriography was carried out (via the existing carotid access or via re-puncture as necessary) to evaluate the patency of the thoracoabdominal aorta and its main branches, including the celiac trunk, superior mesenteric artery (SMA), renal arteries, and lumbar arteries. Immediately following this final arteriography, the animals were euthanized by an intravenous overdose of anesthetics.

Blood samples were obtained pre-procedure and post-procedure (i.e., prior to euthanasia) for serum analysis of creatine kinase (CK), lactate, and amylase. Gastrointestinal segments (stomach, small intestine, and colon) were collected en bloc and fixed in 10% formalin for histopathological analysis. Paraffin-embedded samples were stained with hematoxylin and eosin (HE) and evaluated by a pathologist blinded to group allocation, using the Park/Chiu classification^10^.

Statistical analysis was performed using GraphPad Prism version 8.0. Descriptive statistics included mean, median, and standard deviation. Mann-Whitney’s U and Fisher’s exact tests were employed for group comparisons, with significance defined as *p* < 0.05.

## Results

One animal in the G3 died six hours postoperatively, possibly due to malignant hyperthermia. Thus, nine animals in G3 and five in G0 were included in the final analysis.

### Pre-euthanasia arteriography findings

A final arteriography was performed under anesthesia on the eighth day, immediately before euthanasia. It revealed that all major visceral branches–including the celiac trunk, the SMA, and the renal arteries–remained patent in both G3 and G0. There were no signs of occlusion or significant stenosis at the origins of these vessels. The patency of the mesenteric arteries corroborates the histological findings of minimal gastrointestinal injury and supports the conclusion that the triple stent configuration does not compromise visceral perfusion.

### Occurrence of gastrointestinal ischemia

The occurrence of gastrointestinal ischemia was evaluated in both groups. In G3, ischemic lesions were identified in 88.9% of animals, with lesions predominantly located in the colon (89%), followed by the small intestine (22%) and the stomach (11%). In G0, ischemic lesions were present in 60% of animals, primarily affecting the stomach (40%) and colon (60%). Table 1 summarizes these findings.

**Table 1:** Distribution of gastrointestinal ischemia in the triple stent and control groups.

Group	Organ	Number of animals (%) with ischemia
Stent (n = 9)	Stomach	1 (11)
	Small intestine	2 (22)
Control (n = 5)	Colon	8 (89)
	Stomach	2 (40)
	Small intestine	2 (40)
	Colon	3 (60)

Source: Elaborated by the authors.

**Figure 2:**
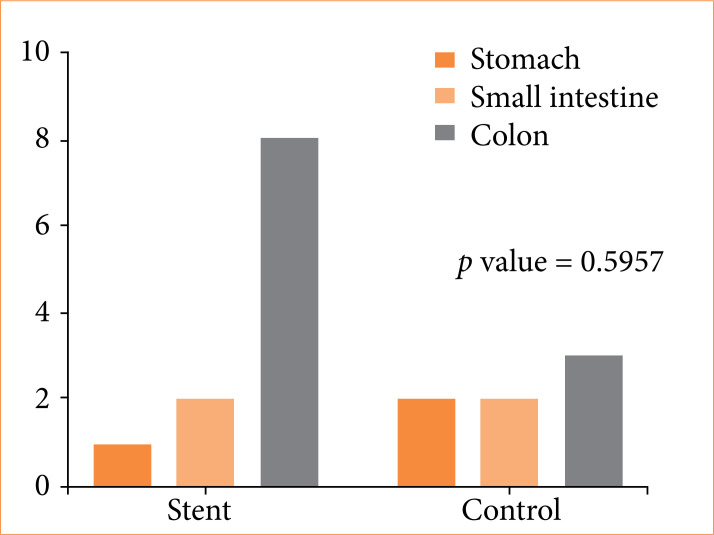
Total frequency of histological lesions.

Source: Elaborated by the authors.

Figure 2 shows the frequency of ischemic and non-ischemic lesions in both groups. No significant difference was found in the frequency of gastrointestinal ischemia between G3 and G0 (*p* = 0.5055).

**Figure 3:**
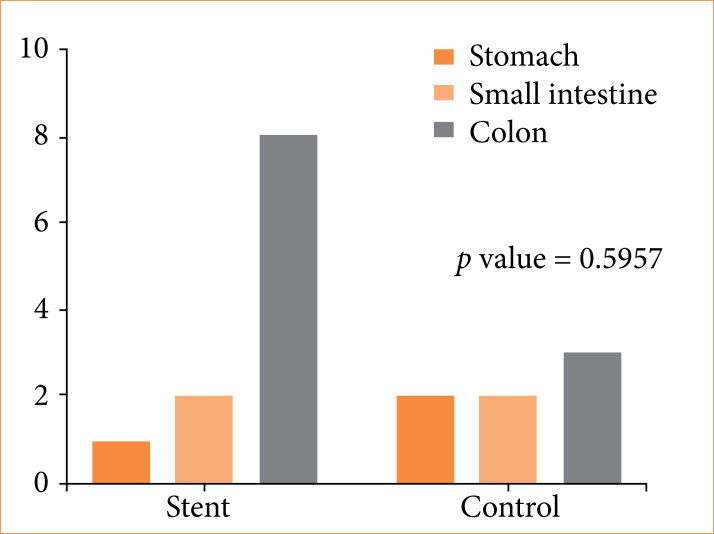
Distribution of histological lesions by organ.

Source: Elaborated by the authors.

Figure 3 illustrates the distribution of ischemic lesions according to organ involvement. The colon showed the highest frequency of lesions in G3, whereas the stomach was more frequently affected in G0. There was no statistically significant difference between groups (p = 0.5957).

### Histological grading

**Figure 4:**
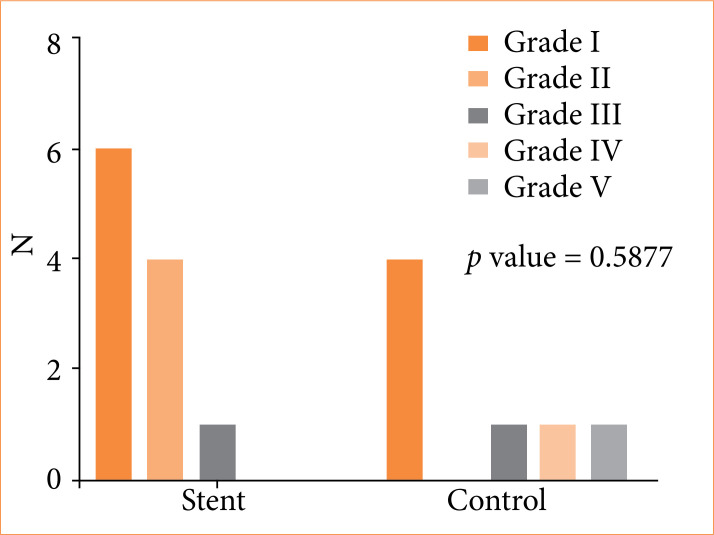
Distribution of histological lesions by grade.

Source: Elaborated by the authors.

Histological lesions were graded from 1 to 5 based on the Park/Chiu classification. The most common lesion grade in G3 was grade 1, with isolated cases of grade-2 and 3 lesions. In G0, higher-grade lesions, including grades 4 and 5, were observed predominantly in the stomach. Figure 4 presents the distribution of lesions by grade, with no significant difference between groups (*p* = 0.5877).

### Biochemical analysis

Serum CK, lactate, and amylase levels were evaluated before and after the procedure. No significant differences were found between groups. Table 2 presents the mean values of the biochemical parameters analyzed.

**Table 2:** Laboratory results (mean values).

Parameter	Triple stent (G3)	Control group (G0)	Reference range^11^
Pre-procedure amylase (U/L)	1,382	895	813–4626
Post-procedure amylase (U/L)	1,198	892	
Pre-procedure creatine kinase (U/L)	1,330	600	61–1251
Post-procedure creatine kinase (U/L)	1,402	745	
Pre-procedure lactate (mmol/L)	26	23	≤ 2.5
Post-procedure lactate (mmol/L)	22	45	

Source: Elaborated by the authors.

**Figure 5:**
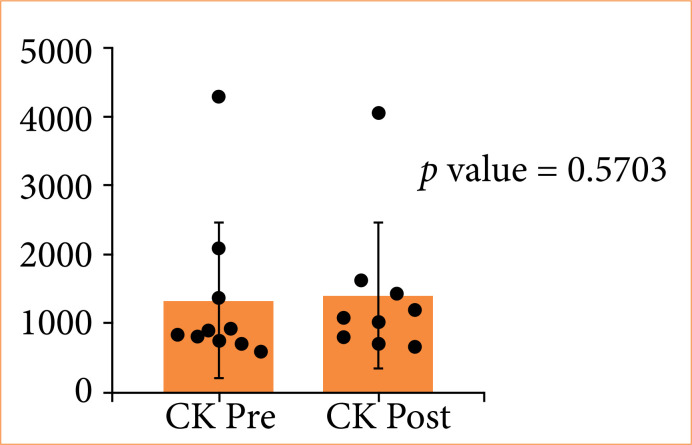
Distribution of creatine kinase (CK) levels pre- and post-procedure.

Source: Elaborated by the authors.

Figure 5 shows CK levels pre- and post-procedure, with similar distributions in both groups (*p* = 0.5703).

**Figure 6:**
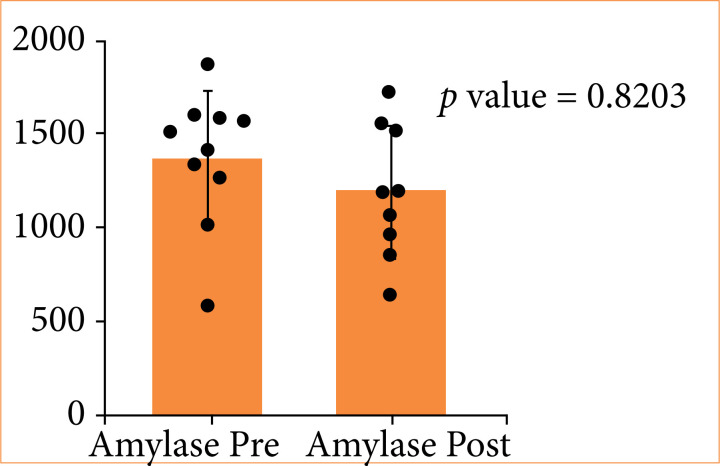
Distribution of amylase levels pre- and post-procedure in triple stent group.

Source: Elaborated by the authors.

**Figure 7:**
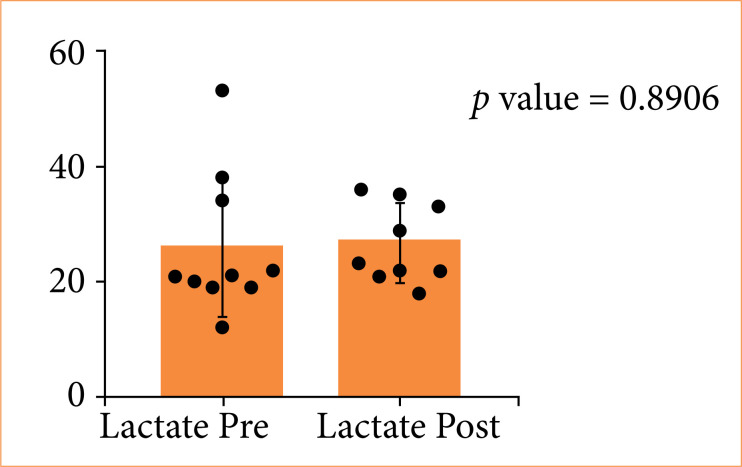
Distribution of lactate levels pre- and post-procedure in triple stent group.

Source: Elaborated by the authors.

In G3, serum amylase levels showed a slight decrease post-procedure, but this was not statistically significant (*p* = 0.8203) (Fig. 6). Lactate levels remained stable, indicating no evidence of metabolic acidosis or systemic ischemia (Fig. 7, *p* = 0.8906).

## Discussion

This study aimed to assess whether triple stent interposition in the thoracoabdominal aorta leads to any significant increase in the frequency or severity of gastrointestinal ischemic injury compared to a G0, which only underwent arteriography. The results demonstrated no significant difference between groups, suggesting that the use of a triple stent is safe regarding gastrointestinal perfusion.

The final arteriography performed prior to euthanasia indicated that none of the stented animals experienced occlusion or significant stenosis of the major visceral branches. This corroborates previous findings in which carefully selected stent designs and deliberate overlaps have been shown to maintain adequate flow in essential side branches, even during extensive thoracoabdominal coverage. Moreover, ensuring patency of the superior mesenteric artery is crucial, considering the large portion of the gastrointestinal tract it supplies.

The histological injury observed was predominantly grade 1, according to the Park/Chiu classification, characterized by subepithelial edema without significant necrosis or transmural infarction. This finding aligns with previous studies reporting low-grade lesions after endovascular procedures, indicating that such changes are reversible and clinically insignificant^12^^,^ 13.

The absence of higher-grade lesions in the triple stent group suggests that the overlapping of stents does not compromise the perfusion of mesenteric vessels. Studies have shown that the implantation of multiple stents does not necessarily increase the risk of visceral ischemia when appropriate techniques are employed^13^^,^ 14.

Although there was no statistically significant difference between the groups, the presence of ischemic lesions in both suggests that other factors may contribute to gastrointestinal injury, such as surgical manipulation, anesthesia, and transient hemodynamic alterations^15^. Additionally, the higher frequency of lesions in the colon may be attributed to its greater susceptibility to hypoperfusion due to the less redundant vascular anatomy of this region^16^.

The limitations of this study include the small sample size, which may limit the statistical power to detect subtle differences between groups. The eight-day follow-up period, although adequate to assess the initial histological response, may not be sufficient to detect late complications or vascular remodeling processes^17^.

Future studies should consider larger samples and longer follow-up periods to fully evaluate the safety and efficacy of triple stent interposition. Furthermore, the use of animal models with comorbidities like patients with thoracoabdominal aortic aneurysms could provide insights more applicable to clinical practice.

## Conclusion

The interposition of a triple stent did not result in a significant increase in gastrointestinal histological injury compared to the control group in the thoracoabdominal aorta of pigs. These findings suggest that the triple stent technique is a safe endovascular strategy for treating complex aortic pathologies without compromising gastrointestinal integrity or perfusion. Additional studies with a larger number of animals and longer follow-up periods are necessary to confirm these results and establish their clinical applicability in humans.

## Data Availability

All data generated or analyzed during this study are available from the corresponding author upon reasonable request.
